# Essential Oils of Aromatic Plants with Antibacterial, Anti-Biofilm and Anti-Quorum Sensing Activities against Pathogenic Bacteria

**DOI:** 10.3390/antibiotics9040147

**Published:** 2020-03-30

**Authors:** Marlon Cáceres, William Hidalgo, Elena Stashenko, Rodrigo Torres, Claudia Ortiz

**Affiliations:** 1 Escuela de Medicina, Universidad Industrial de Santander, Bucaramanga 680002, Colombia; marlon_caceres08@hotmail.com; 2 Escuela de Química, Universidad Industrial de Santander, Bucaramanga 680002, Colombia; elenastashenko@gmail.com; 3 Laboratorio de Biotecnología CEO, Instituto Colombiano del Petróleo, ECOPETROL, Piedecuesta 681012, Santander, Colombia; rodrigo.torres@ecopetrol.com; 4 Escuela de Microbiología y Bioanálisis, Universidad Industrial de Santander, Bucaramanga 680002, Colombia; ortizc@uis.edu.co

**Keywords:** Essential oil, pathogenic bacteria, biofilm, quorum sensing, microbial resistance

## Abstract

Both the ability of bacteria to form biofilms and communicate through quorum sensing allows them to develop different survival or virulence traits that lead to increased bacterial resistance against conventional antibiotic therapy. Here, seventeen essential oils (EOs) were investigated for the antimicrobial, antibiofilm, and anti-quorum sensing activities on *Escherichia. coli* O157:H7, *Escherichia coli* O33, and *Staphylococcus epidermidis* ATCC 12228. All essential oils were isolated from plant material by using hydrodistillation and analyzed by GC-MS. The antimicrobial activity was performed by using the microdilution technique. Subinhibitory concentrations of each EO were assayed for biofilm inhibition in both bacterial strains. Quantification of violacein in *Chromobacterium violaceum* CV026 was performed for the anti-quorum sensing activity. The cytotoxicity activity of the EOs was evaluated on Vero cell line by using MTT method. Thymol-carvacrol-chemotype (I and II) oils from *Lippia origanoides* and *Thymus vulgaris* oil exhibited the higher antimicrobial activity with MIC values of 0.37–0.75 mg/mL. In addition, these EOs strongly inhibited the biofilm formation and violacein (QS) production in a concentration-dependent manner, highlighting thymol-carvacrol-chemotype (II) oil as the best candidate for further studies in antibiotic design and development against bacterial resistance.

## 1. Introduction

The antimicrobial resistance is, currently, an urgent threat to global public health. The poor set of antibiotics available in the market, its indiscriminate abuse in the treatments, and the slow rate of new therapeutic agents have led to an antibiotic resistant crisis during the last decades [1]. In this context, biofilms, a consortium of cell bacteria with a complex matrix of DNA, proteins, and polysaccharides, are among the most relevant clinical importance, as they protect the microorganism by allowing them survive hostile environments, hinder antibiotic uptake, and represent more than 80% of the microbial infections worldwide [2,3]. The formation of bacterial biofilms is well known for their physical and biological properties that confers resistance against antibiotics, and therefore it is one of the major challenges in the current antibiotic therapy [4,5].

*Escherichia coli* and *Staphylococcus epidermidis* are among the bacterial species of high interest in clinical research. *E. coli* O157:H7 is a Gram-negative foodborne pathogen that produce Shiga toxins, and it is responsible of causing diseases such as hemorrhagic colitis, hemolytic uremic syndrome, and the fatal thrombotic thrombocytopenic purpura [6,7]. *E. coli* O157:H7 is able to form biofilms on biotic and abiotic surfaces such as stainless steel sheet, polymers, glass, and plant tissues if the appropriate conditions are given [8]. On the other hand, *S. epidermidis* is a Gram-positive opportunistic pathogen that causes biofilm to grow on intravascular devices placed within the body and fracture fixation infections [9]. The biofilm confers antibiotic resistance and induces a decrease in host responses, leading towards severe conditions such as bacteremia, sepsis, and even the death [10]. Approximately 80% of the most common infections during medical implant procedures such as joint prostheses, cardiac pacemakers, and central venous catheters are caused by *S. epidermidis* [11].

Quorum sensing (QS), a mechanism of bacterial communication by using diffusible molecules known as autoinducers, plays an important role in the gene expression induction to control the cell behaviors such as bioluminescence, virulent factors secretion, biofilm development, and survival to antimicrobial agents [12,13]. Autoinducers are studied under three different classes based on their structure and specific function: Acyl homoserine lactones (AHLs) are responsible for facilitating cell signaling in Gram-negative bacteria, autoinducing peptides (AIP) are the molecules responsible for bacterial communication in Gram-positive bacteria, and autoinducer-2 (AI-2) represents furanones that allow communication in Gram-negative and Gram-positive bacteria [14,15]. Therefore, searching for new therapeutic candidates that interfere with bacterial QS-production has become an urgent and interesting research topic to try to counteract the bacterial resistance [16].

The essential oils (EOs), a mixture of a large and diverse class of terpenoid and phenolic compounds isolated from aromatic plants, have been of such a great interest during the last decades for exhibiting broad biological properties; among them, antibacterial, antiparasitic, antifungal, and antiviral properties have been reported [17,18]. In addition, previous studies have also demonstrated the potential activity of EOs as antineoplastic, anti-inflammatory, allelopathic, antioxidant, insecticidal, and repellent, making them excellent candidates for new natural drugs discovery [19,20,21]. Thus, in this study, *E. coli* and *S. epidermidis* were both selected for studying the antimicrobial, antibiofilm and anti-QS activities of seventeen essential oils derived from *Lippia origanoides*, *Thymus vulgaris*, *Lippia alba*, *Cymbopogon martini*, *Cymbopogon flexuosus*, *Rosmarinus officinales*, *Salvia officinales*, *Swinglea glutinosa*, *Tagetes lucida*, *Satureja viminea*, *Cananga odorata*, *Citrus sinensis*, and *Elettaria cardamomum*.

## 2. Results

### 2.1. Chemical Analysis of the Essential Oils (EOs)

The major metabolites present in each EO were identified and quantified by GC-MS analysis (Table 1).

### 2.2. Antibacterial Activity of EOs

**Figure 1:**
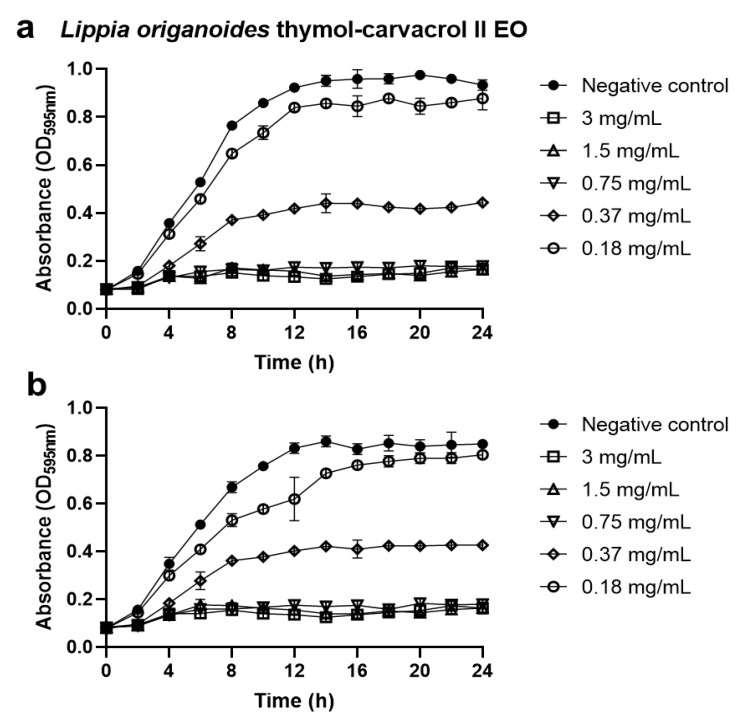
Antibacterial activity of the essential oil “thymol-carvacrol-chemotype (II)” from *Lippia origanoides* against the growth rate of *E. coli* O157:H7 (**a**) and *S. epidermidis* ATCC 12228 (**b**). Data are presented as the mean ± SD of absorbance measured at 595 nm.

The minimal inhibitory concentration (MIC_50_) and minimal bactericidal concentration (MBC) results are summarized in Table 2. The results clearly showed the potential biological activity of several EOs against most of the bacterial species tested. The thymol-carvacrol-chemotype (I and II) oils from *Lippia origanoides* exhibited the highest antimicrobial activity followed by *Thymus vulgaris* oil (Figure 1). In contrast, carvona- and citral-chemotype oils from *Lippia alba*, *Cymbopogon nardus* oil, *Swinglea glutinosa* oil, and *Cananga odorata* oil did not show significant results.

### 2.3. Anti-Biofilms Activity of the Essential Oils

**Figure 2:**
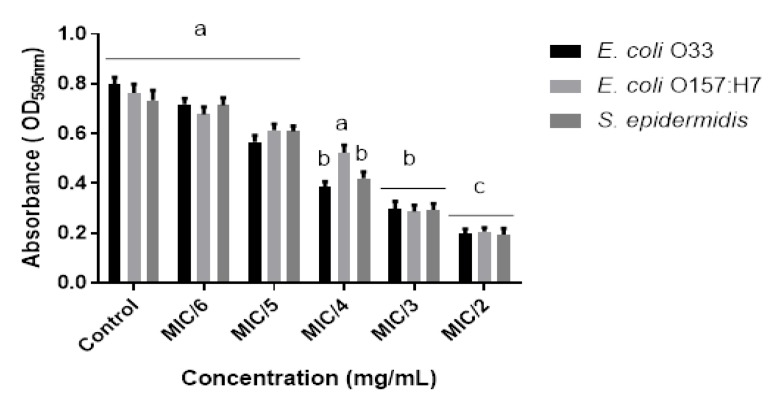
Biofilm inhibition activity of thymol-carvacrol-chemotype (II) oil from *Lippia origanoides* (at different sub-lethal MIC_50_ concentrations) against *E. coli* O33 (black bars), *E. coli* O157:H7 (light-gray bars), and *S. epidermidis* (dark-gray bars). Data are presented as mean ± SD of absorbance (at 595 nm). ANOVA (*p* < 0.05) was performed, followed by Tukey’s Test. Different letters indicate significant differences between the test groups.

**Figure 3:**
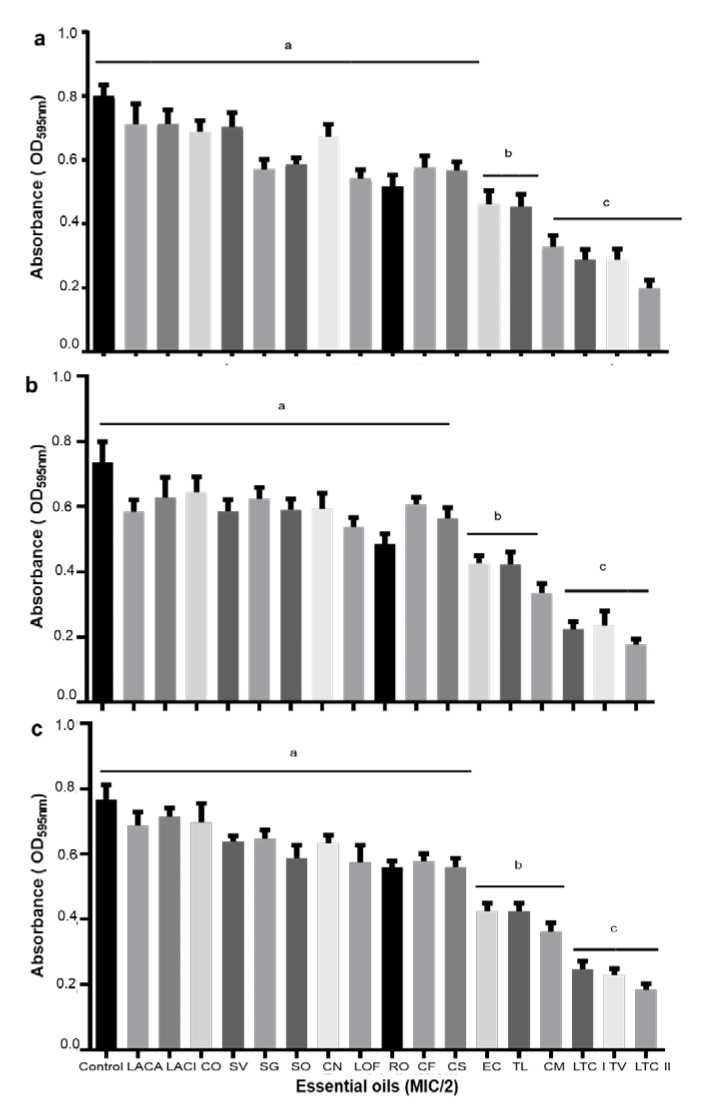
Anti-biofilm activity of different essential oils (at MIC_50_/2 concentration) on bacterial strains. *E. coli* O157:H7 (**a**), *E. coli* O33 (**b**), and *S. epidermidis* ATCC 12228 (**c**). *Lippia alba* carvone chemotype (LACA), *Lippia alba* citral chemotype (LACI), *Cananga odorata* (CO), *Satureja viminea* (SV), *Swinglea glutinosa* (SG), *Salvia officinalis* (SO), *Cymbopogon nardus* (CN), *Lippia origanoides* felandrene chemotype (LOF), *Rosmarinus officinalis* (RO), *Cymbopogon flexuosus* (CF), *Citrus sinensis* (CS), *Elettaria cardamomum* (EC), *Tagetes lucida* (TL), *Cymbopogon martini* (CM), *Lippia origanoides* thymol-carvacrol chemotype (I) (LTC I), *Thymus vulgaris* (TV), and *Lippia origanoides* thymol-carvacrol chemotype (II) (LTCII). The bars on the graph represent mean ± SD of biofilm inhibition of triplicate experiments. The ANOVA test showed a statically significant difference relative to control (*p* < 0.05). with a significance level of 95% and a mean comparison Tukey’s test with an α error of 0.05. Different letters indicate significant differences between the test groups.

A high inhibitory effect on biofilm formation was observed for most of the essential oils tested. Interestingly, thymol-carvacrol-chemotype (II) oil from *Lippia origanoides* exhibited the most detrimental activity on biofilm formation against all bacterial tested. A biofilm reduction of 75, 73, and 74% on *E. coli* O33, *E. coli* O157:H7, and *S. epidermidis* ATCC12228, respectively, was determined for this oil (Figure 2). Similar results were found for thymol-carvacrol-chemotype (I) oil from *Lippia origanoides*, *Thymus vulgaris* oil, and *Cymbopogon martini* oil against *E. coli* O157:H7, *E. coli* O33, and *S. epidermidis* (Figure 3).

### 2.4. Anti-Quorum (AQ) Sensing Activity

**Figure 4:**
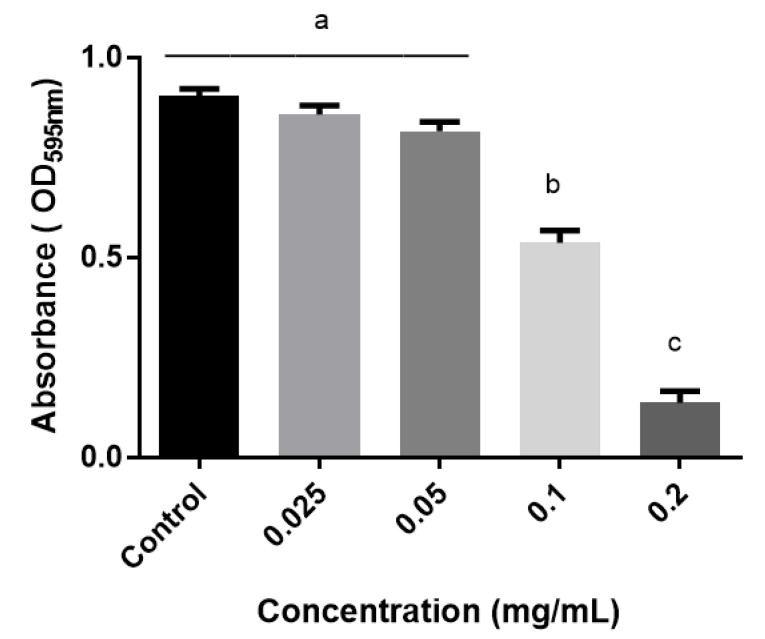
Violacein production in CVO26 bacterial strain during treatment with thymol-carvacrol-chemotype (II) oil from *Lippia origanoides*. Data are presented as mean ± SD of absorbance (at 595 nm). ANOVA (*p* < 0.05) was performed, followed by Tukey’s Test. Different letters indicate significant differences between the test groups.

MIC of the EOs against the reference bacterial strain *Chromobacterium violaceum* CVO26 ranged from 0.18 mg/mL to 3.0 mg/mL (Table 1). A series of sub-MICs concentrations were selected for violacein production screening using CV026 strain. A significant reduction of 88.2% in the pigment production (violacein) was observed with the thymol-carvacrol-chemotype (II) oil from *Lippia origanoides* (Figure 4). In a lesser extent, essential oils derived from thymol-carvacrol chemotype (I) from *Lippia origanoides* and *Elettaria cardamomum* exhibited a reduction of 70% and 65%, respectively. Bacterial viability of CVO26 was also assessed for both control and treated samples with the EOs and the results, in terms of colony-forming units/mL (CFU/mL), did not show significant differences between both groups. Therefore, the reduction in violacein production was attributed to the biological effect caused by the EO.

### 2.5. Cellular Viability Assays

The EO’s exhibiting the highest anti-biofilm activity were selected for cellular viability assays on Vero cell line. Cytotoxicity concentration 50% (CC_50_) values were determined so that the results are reported in Table 3.

Selectivity index (SI) was determined as the logarithm ratio of CC_50_ and the MIC value for each microorganism (SI = log [CC_50_]/[MIC]) [22,23]. Positive values represent elevated selectivity against microorganisms, whereas negative values represent high toxicity to Vero cells. Thymol-carvacrol-chemotype (II) oil from *Lippia origanoides* and *Thymus vulgaris* oil had positive SI value (selectivity index) in all concentrations tested. Therefore, it means that the above essential oils were more selective against bacteria rather than Vero cells. In contrast, thymol-carvacrol chemotype (I) oil from *Lippia origanoides* and *Cymbopogon martini* oil did show negative SI values, indicating a higher toxicity towards Vero cells.

### 2.6. Scanning Electron Microscope (SEM) Analysis

**Figure 5:**
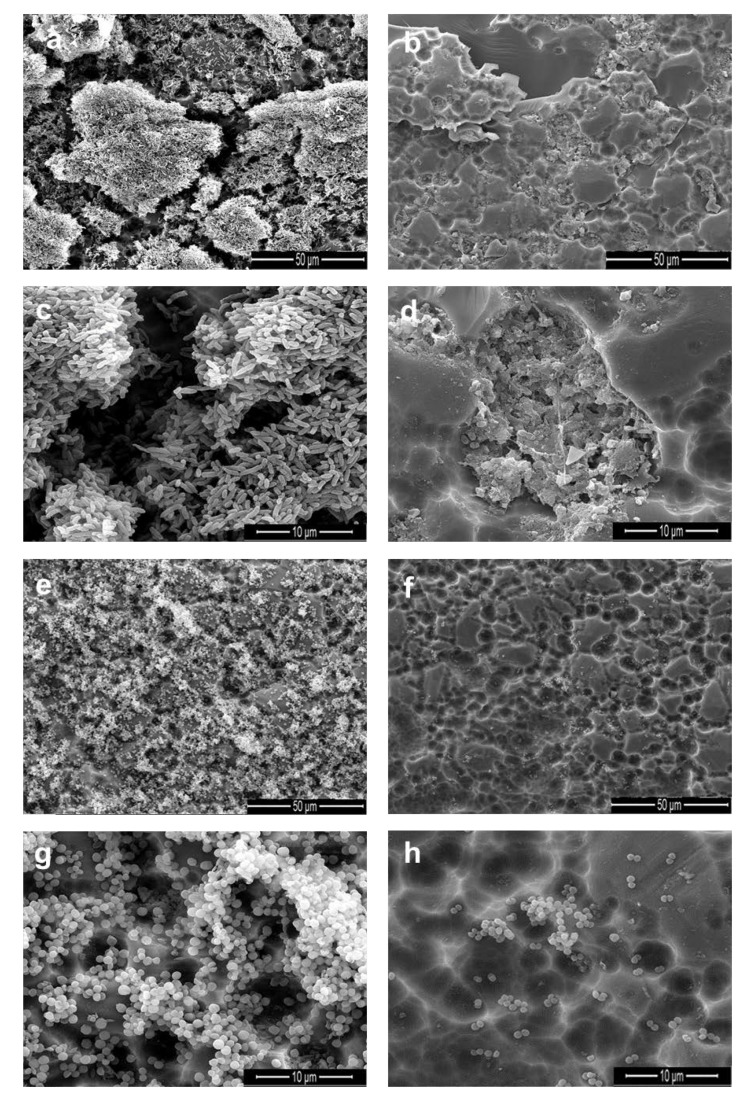
SEM images of biofilm formation in *E. coli* O157:H7 (**a**,**c**) and *S. epidermidis* (**e**,**g**). Biofilm disruption on *E. coli* O157:H7 (**b**,**d**) and *S. epidermidis* (**f**,**h**) after EO treatment. SEM images were recorded at 2000× and 8000× magnification.

The SEM analysis allowed visualizing the biofilms layers formed by *E. coli* O157:H7 (Figure 5a,c) and *S. epidermidis* ATCC12228 (Figure 5e,g) after growing for 24 h and without EO treatment (control). Here, once exposed to thymol-carvacrol-chemotype (II) oil (treatment), a disruption of the cellular and structural integrity of the biofilm was observed (Figure 5b,d,f,h). These results are in accordance with the inhibition biofilm assays showed previously.

## 3. Discussion

The plant kingdom constitutes a priceless source of natural products with potential benefits not only for the plants and the ecosystems, but also for the human being as well as research and the industry worldwide. Essential oils, a mixture of a diverse number of liposoluble metabolites, have attracted attention in the last decades for their numerous biological applications, among them, their antimicrobial properties against bacterial such as *E. coli* and *S. epidermidis* [24].

In this study, seventeen essential oils were used for exploring their antimicrobial and anti-biofilm activities against *E. coli* O157:H7 and *S. epidermidis*, being both bacterial species highly important in the medical field. *E. coli* causes diseases such as hemorrhagic colitis, hemolytic uremic syndrome and thrombotic thrombocytopenic purpura, whereas *S. epidermidis* is associated to post-surgery infections of medical implants.

The essential oils assessed did show strong bacterial growth inhibition on *E.coli* O157:H7, *E. coli* O33, and *S. epidermidis* ATCC 12228 as it was described above by the MIC and MBC results (Table 2). Thymol-carvacrol chemotype (I and II) oils from *Lippia origanoides* along with *Thymus vulgaris* oil were found to be the most effective antimicrobials, exhibiting MICs values in the range of 0.37 to 0.75 mg/mL. The major phenolic compounds identified in *Lippia origanoides* oils, carvacrol, and thymol, are well recognized for exerting higher biological activity in comparison with other components such as limonene, geraniol, citronellal, and 1,8-cineol, the major metabolites of some essential oils [25,26,27]. On the other hand, the metabolites γ-terpinene and p-cymene, although they were found in relatively moderate quantities in *T. vulgaris*, to date, they have not been associated with antimicrobial properties [28]. Proof of the poor biological activity of p-cymene has previously reported in trials against *Mycobacterium tuberculosis*, *S. aureus* (MRSA), *Haemophilus influenzae*, Salmonella *enterica* serovar typhi, and *Vibrio cholerae*; nonetheless, in mixture with other metabolites as carvacrol and thymol, it seems to increase the bacterial inhibition likely by a synergistic mechanism [29,30,31].

Note that although the action mechanism of thymol and carvacrol has not been completely elucidated, it appears to act through a disruption of the cell membrane, which leads to increased cell permeability, membrane potential decay, dissipation of pH gradients, intracellular nutrient leaking, and subsequent cell death [32,33]. Picone and co-workers [34] also reported a strong enzymatic inhibition, particularly in the glycolysis and Krebs cycle pathways, when *E. coli* was treated with carvacrol; however, this toxic effect could be reduced when glucose was added to the culture medium. On the other hand, thymol seems to have a greater activity against gram-positive than gram-negative bacteria, in part, due to lipid disturbance on the plasma membrane caused by this metabolite [35].

Bacterial biofilm, a matrix of extracellular polymeric substances, has become as one of the main factors on the rapid emergence of resistant bacteria to conventional antibiotics. Therefore, new therapeutic strategies are currently needed to counter the negative impact caused by the bacteria resistance to antibiotics. In this regard, essential oils with antibiofilm properties could be a priceless and safe alternative against the emergence bacterial resistance. In this study, the effect of EOs on biofilm formation from *E. coli* O157:H7, *E. coli* O33, and *S. epidermidis* ATCC 12228 was investigated. From the results, it was noteworthy the high anti-biofilm activity exhibited by thymol-carvacrol-chemotype (II) oil from *Lippia origanoides*, which, in fact, did not affect the rate growth of planktonic bacteria.

Kim and co-workers [36] investigated the effect of 83 essential oils on biofilm formation in *E. coli* O157:H7 bacterial strain. Cinnamon bark, bay, clove, and pimento berry oils exhibited the highest anti-biofilm activity, inhibiting by more than 75% when a concentration of 0,005% (*v/v*) was tested. The plant extract of *Carex dimorpholepis* has also been reported by possess anti-biofilm properties on *E. coli* O157:H7 [37].

Previous studies have demonstrated the anti-biofilm activity of monoterpenoids both on Gram-positive and Gram-negative bacteria during the first stages of biofilm development [38,39,40]. Particularly, carvacrol seems to cause a decrease in flagellar development of the bacterial cell, leading to loss of cell motility and affecting biofilm development on several pathogenic bacteria [41]. Our results suggest that thymol-carvacrol-chemotype I and II oils from *Lippia origanoides* are potential inhibitors of the biofilm development on *E. coli* and *S. epidermidis.* For the last case, interestingly, it was observed a higher inhibitory effect of the EO on the biofilm development, which in fact, was even higher than those reported by other authors [42]. Here, the inhibition of poly-N-acetylglucosamine polymers biosynthesis, the major components of the biofilm structure in *S. epidermidis*, could be a plausible mechanism of the effect caused by the essential oil.

Therefore, the anti-QS activity of the EOs used in our study was also explored by using *C. violaceum* CV026 as biological model. All essential oils did not exhibit anti-QS activity except for thymol-carvacrol chemotype (I and II) from *Lippia origanoides* and *Elettaria cardamomum*, which significantly inhibited the violacein production at 0.20, 0.75, and 1.00 mg/mL, respectively. Although the action mechanism of the EOs is not completely understood, our results suggest that they could act through the inhibition in the production of AHL or by blocking the cell–cell communication system. Cervantes and coworkers did report a poor anti-QS activity of the essential oils from *Lippia origanoides* at the concentrations tested (2.5–25.0 µg/mL). However, note that thymol and carvacrol metabolites were present in low concentrations in such EO, and thereby it highlights the importance of these metabolites as the ones responsible for the anti-QS activity [43]. In addition, carvacrol oil in a range of 0.1 to 0.4 mM, has been reported for strongly inhibit violacein production, highlighting the importance of this metabolite in bacterial QS [24].

On the other hand, Ahmad and coworkers did study the anti-QS effect of some metabolites present in the EOs. Among their findings, the metabolite ρ-cimeno exhibited a higher activity than its counterpart carvacrol and thymol [44]. Therefore, the anti-QS activity of the thymol-carvacrol-chemotype (I and II) oils from *L. origanoides* could be the result of a synergistic mechanism among the diverse metabolites present which contribute to potentiate the biological activity. Nevertheless, further studies are needed to understand the biological role of each metabolite present in the EOs as inhibitors of biosynthetic pathways involved in bacterial resistant and pathogenesis.

The essential oils represent an excellent alternative to be used as antibiotics against resistant bacterial species. In this sense, they should meet health safety for inclusion as therapeutic treatments. Therefore, a preliminary study of EOs cytotoxicity was performed in Vero cell line. Interestingly, the selectivity index (SI) found for thymol-carvacrol-chemotype (I and II) from *L. origanoides* had a higher affinity towards the bacterial species assessed rather than Vero cells. Overall, this result along with the high anti-biofilm and -QS activities found for these essential oils, position them as promising natural products in the development of new and better therapeutic strategies to the emergence clinical issues.

## 4. Materials and Methods

The plants used in this study were harvested from the experimental lots located in the Agroindustrial Pilot Complex of CENIVAM (National Center for Research on Agro-Industrialization of Tropical Medicinal Aromatic Plants), at Universidad Industrial de Santander (Bucaramanga, Colombia). The taxonomic characterization of the plants was carried out in the Institute of Natural Sciences of the Universidad Nacional of Colombia (Bogotá, Colombia). *Staphylococcus epidermidis* ATCC 12228 and *Escherichia coli* O33 strains were donated by the School of Microbiology of Universidad Industrial de Santander. *Escherichia coli* O157:H7 was provided by Pontifical Xavierian University (Bucaramanga, Colombia). *Chromobacterium violaceum* CV026 was gently donated by Dra. Nohora Rueda from Universidad de Santander—UDES.

### 4.1. Essential Oils Analysis

Essential oils were extracted by microwave-assisted hydrodistillation (MWHD) in a Clevenger-type distillation equipment adapted to a heating system in a Samsung MS-1242zk domestic microwave (Seúl, Korea oven with an output power of 1600 W and 2.4 GHz radiation frequency. The plants (200 g) suspended in water (300 mL) were placed in a 2 L balloon, which was connected to a glass equipment, type Clevenger, with a Dean–Stark distillation reservoir. The plant sample was heated by microwave irradiation for 45 min (3 × 15min, consecutives). The essential oil obtained was dried with anhydrous sodium sulfate, weighed and store in an amber bottle at 4 °C until further analysis. All extractions were made in triplicate [45].

### 4.2. GC-MS Analysis of Essential Oil

The essential oil analysis was carried out using an Agilent Technologies 6890N Series Network System (Palo alto, California, USA) coupled to an Agilent Technologies MSD 5975 *Inert* XL mass-selective detector. An apolar capillary column DB-5MS (60 m × 0.25 mm i.d × 0.25 µm, d_f_), with a stationary phase of 5% phenyl-poly-(dimethylsiloxane), and a polar capillary column DB-WAX (60 m × 0.25 mm i.d × 0.25 µm, d_f_) with stationary phase of poly(ethylene glycol) were used. Helium was used as carrier gas (99.995%), with a constant volumetric flow of 1 mL/min. The oven temperature was programmed from 45 °C (5 min) to 150 °C (3 min) at 3 °C/min, then up to 220 °C (5 min), at 4 °C/min.

The mass spectra were obtained by electron impact (EI) with energy of 70 eV. The temperature of the ionization chamber and the transfer line remained at 230 and 285 °C, respectively. The mass spectra, total ionic currents (TIC), and extracted ion (EIC) were obtained with a quadrupole analyzer, by means of automatic radiofrequency scanning (full scan) in the mass range of *m/z* 40-350 (5.5 spectra/s). The components of the essential oils were identified by comparison of their mass spectra, obtained by GC-MS, and linear retention index (LRI) in the two columns—polar and non-polar—based on the calculated base of the homologous series of n-alkanes C9-C25 and compared with those of different mass spectral bases and data from scientific literature [46,47].

For the confirmatory identification of components in the essential oils, extracts or volatile fractions, the following standard substances, purchased from Sigma-Aldrich were used; Geranyl acetate (98%), aromadendrene (97%), benzyl benzoate (98%), β- caryophyllene (98.5%), 1.8-cineol (99%), ρ-cymene (99%), α-copaene (90%), trans-farnesol (96%), geraniol (98%), hexanal (98%), α-humulene (96%), linalool (97%), (*R*)–(+)–limonene (97%), menthol (99%), (+)–menthone (98.5%), trans-nerolidol (85%), caryophyllene oxide (95%), and α-pinene (98%). All solvents used were HPLC grade.

### 4.3. Determination of Antimicrobial Activity

Antimicrobial activity of EOs was carried out as previously described with some modifications [48]. Briefly, The MIC of essential oils was determined using the broth microdilution method for bacteria, in a 96-well microplate. The essential oils were dissolved in dimethyl sulfoxide (DMSO). Serial dilutions of the essential oils were prepared ranging from 3.0 up to 0.18 mg/mL to a final volume of 100 µL per well. All experiments were conducted with a maximum of 1% (*v/v*) DMSO in solution. One-hundred microliters of bacterial suspension were added to each well to obtain a final inoculum concentration of 4.6 × 10^7^ CFU mL^−1^ and a working volume of 200µL. *E. coli* O157:H7, *E. coli* O33 and CV026 in LB and *Staphylococcus epidermidis* ATCC 12228 in MH were used as growth control. Ofloxacin was used as a positive control. In vitro cultures were incubated at 37 °C with constant agitation during 24 h and the optical density monitored at 595 nm in a Bio-Rad iMark microplate absorbance reader version 1.02.01 (California, USA).

### 4.4. Effect on Biofilm Formation

The essential oils were assessed for their potential to prevent biofilm formation of a biofilm produced by strains *E. coli* O157:H7, *E. coli* O33, and *S. epidermidis* ATCC 12228. Individual wells of sterile polyvinyl chloride (PVC) flat bottomed microtitre plates as described previously with some modifications [42]. Cultures were grown overnight in 3 mL Tryptic Soy Broth (TSB) with 1% glucose, diluted in growth medium to 5 × 10^5^ CFU/mL for *S. epidermidis* ATCC12228, whereas for *E. coli* O157:H7 and *E. coli* O33 Luria Bertani (LB) culture medium was used in the same proportions. One-hundred microliters of the respective culture medium was transferred into the plate in the presence of 100 µL subinhibitory concentrations (subMIC) of EOs. 100µL of culture medium was used as control. After incubation for 24 h at 37 °C, the biofilms were washed three times with sterile phosphate buffer saline (PBS pH 7.2) to remove free-floating planktonic bacteria. Biofilms formed by adherent sessile organisms in plate were stained with crystal violet (0.4 % *w/v*).

### 4.5. Violacein Inhibition Assay

Production of violacein pigment by *Chromobacterium violaceum* in the presence or absence of essential oils was quantified spectrophotometrically [49]. Briefly, an inoculum of the bacterium was prepared at 0.1 OD (600 nm) and incubated in 3mL of LB broth supplemented with C6-HSL in an Erlenmeyer flask. One-hundred-and-fifty microliters of prepared dilutions of EOs at different concentrations (0.18, 0.37, 0.75, and 1.5 mg/mL) was added to each experimental assay. One-hundred-and-fifty microliters of water (or DMSO) was used as a control. The flasks were incubated at 28 °C during 24 h.

One microliter of each sample was centrifuged at 13000× *g* for 10 min. The supernatant was discarded, and the pellet solubilized in 1 mL of DMSO. The final solution was vortexed for 30 s to homogenize the violacein and centrifuged at 13000× *g* for 10 min to remove cell debris. The violacein was quantified spectrophotometrically at OD_595_ (UV-1800 Shimadzu, Japan) [50]. Percent inhibition of violacein production in the presence of carvacrol-chemotype oil from *Lippia* was measured as follows, [(OD_control_−OD_treated_)/OD_control_] × 100%. Simultaneously, cell viability of CV026 strain was determined by bacteria viable count. All experiments were carried out with three biological replicates.

### 4.6. Cellular Viability Assays

The EOs were evaluated for their in vitro cytotoxicity on the VERO cell line, a cell linage derived from the kidney of African green monkey (*Cercopithecus aethiops*), which has been used in our research group as a suitable cell model for cytotoxicity studies [51]. The cell line VERO was grown in EMEM culture medium containing 10% fetal bovine serum and gentamicyn (50 µg/mL) at 37 °C in a 5% CO_2_ humidified incubator. The cells were plated (7.5 × 10^3^ cells/well) in 96-well microplate. After overnight incubation, VERO cells were treated with EOs at 0.18, 0.37, 0.75, and 1.5 mg/mL during 48 h. Cell viability was assessed by using MTT method [52]. The absorbance was measured at 540 nm using a microplate reader Multiskan go (Thermo Fisher Scientific, Vantaa-Helsinky, Finland). The results were expressed as a percentage of viable cells in comparison to the control (taken as 100%).

### 4.7. Scanning Electron Microscope Analysis

Scanning electron microscope (SEM) was used to investigate the structural modifications of biofilms after treatment with EOs. Biofilm formation of *Escherichia coli* O157:H7 and *Staphylococcus epidermidis* ATCC 12228 was carried out on glass coupons (1 cm × 2 cm). The selected coupons were rinsed three times with phosphate-buffered saline (PBS; pH 7.2).

The preparation of the samples for electron microscopy was performed as follows: soaking of the sample with 2.5% glutaraldehyde for 2 h at room temperature. The coupons were washed using different solutions of isopropyl alcohol: 5, 15, 25, 50, 75, and 100% for 5 min each rinsing at room temperature.

### 4.8. Data Analysis

All the experiments were performed in triplicates and one-way analysis of variance (ANOVA) was used to analyze the differences among the treatments. In all cases, the level of significance was 0.05. Assumption of normality and equally of variances of data was previously tested using Shapiro–Wilk and Levene’s test, respectively.

## 5. Conclusions

Essential oils represent promising control agents to be used in therapeutic treatments. Here, it was demonstrated the high potential activity of thymol-carvacrol-chemotype (I and II) oils from *L. origanoides*, *Thymus vulgaris*, and *Cymbopogon martini* oils against biofilm development and QS biosynthesis both in *E. coli* and *S. epidermidis* bacterial strains. In addition, these EOs did not display any toxic effect on Vero cell line, which indeed, opens the possibility for being used in reasonable design of new therapeutic strategies against the emerging impact of bacterial resistance.

## Figures and Tables

**Table 1:** Major chemical constituents present in the EOs assessed. Relative amount of each metabolite is reported in percentage (%).

Code	Plant Species	Identified Metabolites
LACA	*Lippia alba*	Limonene (29%), β-bourbonone (2.4%), germacrene D (12.2%), carvone (31.3%), and piperitenone (1.5%)
LACI	*Lippia alba*	Limonene (3.9%), *trans*-β-caryophyllene (11.8%), neral (15.4%), geranial (18.9%), and geraniol (6.1%)
CN	*Cymbopogon nardus*	Citronellal (11.6%), 2,6-dimethyl-2,6-octadiene (6.1%), β-citronellol (16.9%), and geraniol (17.8%)
CM	*Cymbopogon martini*	*trans*-β-Ocimene (1.9%), linalool (3.2%), geranyl acetate (1.3%), and geraniol (38.7%)
CF	*Cymbopogon flexuosos*	Neral (24.5%), geranial (33%), geraniol (7.9%), and geranyl acetate (0.5%)
LTC I	*Lippia origanoides*	*p*-Cymene (3.7%), thymyl methyl ether (4.6%), *trans*-β-caryophyllene (7.9%), thymol (22.1%), carvacrol (10.7%), and thymyl acetate (3.9%)
LTC II	*Lippia origanoides*	γ-Terpinene (5.2%), *p*-cymene (1.1%), thymol (32,7%), carvacrol (18.8%), and *trans*-β-caryophyllene (6.4%)
LOF	*Lippia origanoides*	α-Phellandrene (5.7%), 1,8-cineole (11.6%), *p*-cymene (5.7%), *trans*-β-caryophyllene (10.4%), and α-humulene (6.2%).
RO	*Rosmarinus offiicinalis*	α-Pinene (12.7%), camphene (7.7%), 1,8-cineole (17.5%), camphor (14.8%), and *trans*-β-caryophyllene (7.8%).
SO	*Salvia officinalis*	1,8-Cineole (5.3%), *trans*-thujone (20.4%), *cis*-thujone (5.8%), camphor (8.5%), and α-humulene (9.8%).
SG	*Swinglea glutinosa*	α-Pinene (2.6%), *trans*-β-caryophyllene (36.6%), germacrene D (15.4%), germacrene B (10.8%), and *trans*-nerolidol (24.0%).
TL	*Tagetes lucida*	Estragole (79.9%) y β-myrcene (0.9%).
TV	*T* *hy* *mus vulgaris*	γ-Terpinene (9.5%), *p*-cymene (20%), linalool (4.7%), *trans*-β-caryophyllene (9.5%), and thymol (23%).
SV	*Satureja viminea*	1-Isopropenyl-4-methyl-1-ciclohexane (24.4%), *trans*-β-caryophyllene (11.8%), pulegone (11.1%), and *cis*-pulegol (7.1%).
CO	*Cananga odorata*	Linalool (11.7%), methyl benzoate (3.7%), benzyl acetate (10.3%), (Z)-cinnamyl acetate (5.4%), and benzyl benzoate (20.8%).
EC	*E. cardamomum*	1,8-Cineole (8.9%), linalool (6.1%), linalyl butyrate (9.9%), α-terpinyl acetate (45.5%), and *cis*-nerolidol (3.1%).
CS	*Citrus sinensis*	Limonene (57.5%), linalool (7.9%), 1-octanol (2.1%), 4-terpineol (1.7%) y valencene (1.6%).

**Table 2:** Minimal inhibitory concentration to inhibit 50% of bacterial population (MIC_50_) and minimal bactericidal concentration (MBC) (mg/mL) determined for the essential oils assessed. Values are means ±SD of triplicate determinations. ANOVA (*p* < 0.05) was performed, followed by Tukey’s Test. Different letters indicate significant differences between the tested groups.

Essential Oil	*E. coli* O33 MIC50—MBC	*E. coli* O157:H7 MIC50—MBC	*S. epidermidis* MIC50—MBC	*cv*026 MIC50—MBC
*Lippia alba* (carvona)	>3–>3	>3–>3	>3–>3	>3–>3
*Lippia alba* (citral)	>3–>3	>3–>3	>3–>3	>3–>3
*cymbopogon nardus*	>3–>3	>3–>3	>3–>3	>3–>3
*Cymbopogon martini*	3 ± 0.22 ^a^–>3	3 ± 0.14 ^a^–>3	3 ± 0.12 ^a^–>3	1,5 ± 0,12 ^b^–3 ± 0.24 ^a^
*Cymbopogon flexuosus*	>3–>3	>3–>3	>3–>3	3 ± 0.21 ^a^–>3
*Lippia origanoides* (thymol-carvacrol I)	0.75 ± 0.14 ^b^–1.5 ± 0.14 ^b^	0.75 ± 0.10 ^b^ -1.5 ± 0.22 ^b^	0.37 ± 0.04 ^c^–0.75 ± 0.02 ^b^	0.75 ± 0.03 ^b^–0.75 ± 0.02 ^b^
*Lippia origanoides* (thymol-carvacrol II)	0.37 ± 0.03 ^c^–0.75 ± 0.02 ^b^	0.75 ± 0.03 ^b^–0.75 ± 0.03 ^b^	0.37 ± 0.03 ^c^–0.75 ± 0.04 ^b^	0.37 ± 0.05 ^c^–0.75 ± 0.02 ^b^
*Lippia origanoides* (felandreno)	3 ± 0.22 ^a^–>3	>3–>3	3 ± 0.31 ^a^–>3	3 ± 0.24 ^a^–3
*Rosmarinus officinalis*	>3–>3	>3–>3	>3–>3	3 ± 0.26 ^a^–>3
*Salvia officinalis*	>3–>3	>3–>3	>3–>3	3 ± 0.28 ^a^–>3
*Swinglea glutinosa*	>3–>3	>3–>3	>3–>3	>3–>3
*Tagetes lucida*	3 ± 0.23 ^a^–>3	3 ± 0.21 ^a^–>3	3 ± 0.24 ^a^- >3	1.5 ± 0.16 ^b^–3 ± 0.24 ^a^
*Thymus vulgaris*	0.75 ± 0.01 ^b^ –1.5 ± 0.12 ^b^	0.75 ± 0.02 ^b^–1.5 ± 0.12 ^b^	0.75 ± 0.10 ^b^–0.75 ± 0.12 ^b^	0.37 ± 0.05 ^c^–0.75± 0.14 ^b^
*Satureja viminea*	>3–>3	>3–>3	>3–>3	3 ± 0.22 ^a^–>3
*Cananga odorata*	>3–>3	>3–>3	>3–>3	>3–>3
*Citrus sinensis*	>3–>3	<3–>3	>3–>3	3 ± 0.32 ^a^–>3
*Elettaria cardamomum*	>3–>3	>3–>3	>3–>3	3 ± 0.26 ^a^–>3

**Table 3:** Inhibitory concentration 50 (IC_50_) and selectivity index (SI) values determined for the selected essential oils.

Essential oils	Vero cell line		SI			
	IC_50_ (mg/mL) ± SD ^a^	R_2_^b^	*E. coli* O33	*E. coli* O157:H7	*S. epidermidis*	*CV* 026
*Cymbopogon martini*	0,86 ± 0.12	0.97	−0.54	−0.54	−0.54	−0.24
*Lippia origanoides* thymol-carvacrol (I)	0.48 ± 0.02	0.99	−0.19	−0.19	−0.19	−0.19
*Lippia origanoides* thymol-carvacrol (II)	0.83 ± 0.02	0.98	0.35	0.04	0.04	0.35
*Thymus vulgaris*	1.76 ± 0.36	0.94	0.37	0.37	0.37	0.68

^a^ The minimum dilution of essential oils which is capable of inducing cell death or inhibiting the proliferation of 50% of the cells. ^b^ Linear correlation coefficient.
